# Effect of the Preparation Method on the Properties of Eugenol-Doped Titanium Dioxide (TiO_2_) Sol-Gel Coating on Titanium (Ti) Substrates

**DOI:** 10.3390/gels9080668

**Published:** 2023-08-18

**Authors:** Julia Both, Anita-Petra Fülöp, Gabriella Stefania Szabó, Gabriel Katona, Alexandra Ciorîță, Liana Maria Mureșan

**Affiliations:** 1 Department of Chemical Engineering, Faculty of Chemistry and Chemical Engineering, Babeș-Bolyai University, 11 Arany J. St., 400028 Cluj-Napoca, Romania; 2 Department of Chemistry and Chemical Engineering of Hungarian Line, Faculty of Chemistry and Chemical Engineering, Babeș-Bolyai University, 11 Arany J. St., 400028 Cluj-Napoca, Romania; 3 Department of Molecular Biology and Biotechnology, Electron Microscopy Centre, Faculty of Biology and Geology, Babeș-Bolyai University, 44 Republicii St., 400015 Cluj-Napoca, Romania

**Keywords:** Ti grade 5, Ti implants, titanium dioxide sol-gels coating, eugenol, dip-coating, corrosion resistance, antibacterial activity

## Abstract

The focus of this study was the preparation of sol–gel titanium dioxide (TiO_2_) coatings, by the dip-coating technique, on Ti6Al4V (TiGr5) and specific Ti implant substrates. In order to confer antibacterial properties to the layers, Eugenol was introduced in the coatings in two separate ways: firstly by introducing the Eugenol in the sol (Eug–TiO_2_), and secondly by impregnating into the already deposed TiO_2_ coating (TiO_2_/Eug). Optimization of Eugenol concentration as well as long term were performed in orderboth short- and long-term Eugenol concentration was performed to investigate the prepared samples thoroughly. The samples were investigated by electrochemical impedance spectroscopy (EIS) and potentiodynamic polarization curves (PDP). To investigate their resistance against Gram-negative *Escherichia coli* bacteria, microbiological analysis was performed on coatings prepared on glass substrates. Structural studies (FT-IR analysis, Raman spectroscopy) were performed to confirm Eugenol–TiO_2_ interactions. Coating thicknesses and adhesion were also determined for all samples. The results show that Eug–TiO_2_ presented with improved anticorrosive effects and significant antibacterial properties, compared to the other investigated samples.

## 1. Introduction

Post-operative complications, such as infections as a result of lack of tissue integration, have been a leading reason for failed implants [1]. These complications, by default, cause an economic loss and an inability for implants to be effectively used in medicine, which is why antibacterial coatings, as presented in the present study, have shown great potential to prevent and solve the mentioned issues. 

Titanium and its alloys are among medical biology’s most commonly used metals due to their high biocompatibility, bioavailability, excellent mechanical characteristics, impressive anti-corrosion properties, and adequate osteointegration [2]. Titanium alloys can be grouped into α-type titanium alloys, near-α-type titanium alloys, (α + β)-type titanium alloys, and β-type titanium alloys, based on the respective microstructures [3]. Solid titanium typically is present in one of two allotropic forms: α-titanium (which has a hexagonal structure), which, at 885 °C, transforms into β-titanium with a cubic structure and a central volume. Grade 5 titanium (TiGr5), named Ti6Al4V, with reference to its composition, is the most commonly used medical-grade titanium alloy [4]. In addition to the mentioned factors, it is a reported fact that Ti implants are highly susceptible to bacterial adhesion, as well as the release and accumulation of toxic alloying elements into the organism [5]. While naturally forming titanium (IV) oxide layers act as protective layers, they do not provide long-term anticorrosive effects. 

It has been previously reported that Ti surfaces may be treated with different materials and methods to prevent the aforementioned bacterial adhesion. As such, artificially prepared TiO_2_ layers with a more stable chemical composition and antibacterial properties are used for medical applications to prevent bacterial colonization [6,7]. The high demand for such antibacterial coatings, added to the low cost and low-temperature requirement of the well-known and widely applied sol-gel method [8], by which the presented coatings were prepared, all point to the feasibility of the presently studied systems.

Though TiO_2_ coatings can be obtained by several methods like magnetron sputtering [9], vapor deposition [10], and anodic oxidation [11], the most common preparation method is the sol-gel method. This method has countless advantages, among which mild chemical conditions can be highlighted, but at the same time, due to the prolonged nature of the aging and drying process, the method also requires a lot of attention. It is a method suitable not only for TiO_2_ coating preparation but also for the preparation of TiO_2_ nanoparticles [12,13]. The first step of the mentioned synthesis route is the preparation of the colloidal suspension from alkoxy precursors like titanium tetra-isopropoxide [14,15], tetra butyl-ortho-titanate [11], and titanium ethoxide [16]. Alkoxy groups of these materials hydrolyze and subsequently participate in a condensation reaction. The obtained solid particles of the sol form the network through gelation [17]. The obtained gel structure and porosity depend on the relative rate of hydrolysis and condensation reactions. Furthermore, the porosity of the resulting gel and the arrangement of the mesopores are largely determined by the pH of the system. The annealing temperature is another circumstance that determines the structure and properties of the formed gel. The coatings are sometimes cured at high temperatures (above 600 °C), generating a rutile phase while under this anatase [9]. Though at lower heating temperatures, the formed crystallite has a smaller size [10], it was reported that it has a stronger antibacterial effect [18].

In order to further improve the properties of TiO_2_ coatings, a variety of additives such as nitrogen [19], sulfur [20], boron [21], tantalum [22], copper [23], magnesium [24], zinc [25] and silver [26] can also be introduced in the TiO_2_ coatings in order to achieve the specific characteristics given by each additive. Notable antibacterial effects of several chemical compounds, such as bimetallic nanoparticles [27,28], or essential oils, such as clove oil (Eugenol), have been previously widely discussed in scientific literature. 

**Figure 1:**
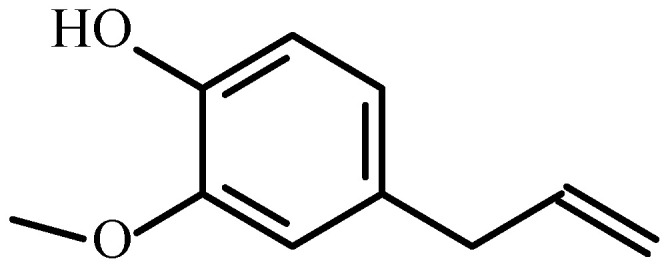
The chemical structure of Eugenol (4-allyl-2-methoxy-phenol).

In the present case, clove oil was chosen as the antibacterial agent to be introduced into the prepared coatings. Eugenol, or 4-allyl-2-methoxy-phenol, with the structure shown in Figure 1, is an antimicrobial, anti-inflammatory, and analgesic substance most commonly used in dentistry [29]. It is a naturally occurring compound found in clove essential oil. Its antimicrobial activity largely depends on its chemical composition and quantity [30]. The anti-corrosive effect of Eugenol was previously tested in the case of aluminum [31,32], titanium-nickel [33], and copper surfaces [34]. The application of antimicrobial, antibacterial, anti-inflammatory, or analgesic agents on the surface of various biomedical tools has become a widely used method of preventing infectious disease [35,36].

Previous studies have shown the successful preparation of efficient antimicrobial TiO_2_ coatings enhanced with silver nitrate [37]. These studies have shown a greater effect of the silver nitrate introduced into the coating system against Gram-positive organisms rather than Gram-negative. Self-disinfecting coatings were also produced, introducing certain inorganic metals or bi-dimensional materials in TiO_2_ and compared regarding their antimicrobial effects in the presence of *Escherichia coli*, methicillin-resistant *Staphylococcus aureus*, *Pseudomonas aeruginosa*, *Bacillus subtilis*, etc. These experiments have concluded that TiO_2_ coatings doped with certain antibacterial agents can be used for dental and orthopedic implants due to their biocompatibility and lack of cytotoxicity [38].

In this context, the present research aimed to prepare Eugenol-loaded TiO_2_ coatings with antimicrobial properties on Ti-based substrates by sol-gelmethod. Our earlier research led to the conclusion that such TiO_2_ coatings present approximately 25% porosity depending on the number of TiO_2_ layers deposited [39]. Previously we experienced several possible ways to introduce a corrosion inhibitor into the coating: firstly, by impregnating the existing pore system by dipping the sample into an inhibitor-containing solution [40,41] and secondly, by directly introducing the inhibitor in the precursor sol [42]. These studies lead to the conclusion that it could be possible to introduce Eugenol in the coatings to improve their properties. 

Consequently, Eugenol was introduced in TiO_2_ layers via two different procedures: (i) by impregnation of TiO_2_ coatings with an alcohol-based, Eugenol-containing solution after TiO_2_ preparation by dip coating (TiO_2_/Eug), or (ii) by introducing Eugenol directly into the precursor sol, before the dip-coating (Eug-TiO_2_). The low heat treatment (150 °C) was chosen in order to obtain a higher antibacterial effect. The prepared coatings were investigated by electrochemical methods such as electrochemical impedance spectroscopy (EIS) and potentiodynamic polarization curves (PDP), which are suitable for the characterization of the permeability of a thin layer [41]. EIS measurements were carried out to determine the prepared coatings’ short- and long-term performance. The antibacterial effect was also determined. Structural analysis was performed by FT-IR analysis to determine the specific bonding between the chemical structures of the additives and TiO_2_. Also, mechanical properties, such as coating thickness and adhesion, were subsequently determined.

## 2. Results and Discussion

### 2.1. Electrochemical Evaluation

#### 2.1.1. Electrochemical Impedance Spectroscopy (EIS) Measurements

First, EIS measurements were performed on TiO_2_ and TiO_2_ layers impregnated with Eugenol in three different concentrations (10^−1^ M, 10^−2^ M, and 10^–3^ M) after TiO_2_ deposition (TiO_2_/Eug). The coatings underwent preliminary electrochemical evaluation, after which the concentration exhibiting the most optimal effects was used to prepare coatings, which were subjected to deeper electrochemical evaluations. The layers in which Eugenol was introduced in the precursor sol before TiO_2_ deposition (Eug-TiO_2_) showed the best effect. Throughout the research, a comparison of the short and long terms electrochemical behaviors of doped and undoped TiO_2_ coatings was performed. Monitorization of coating degradation and active ingredient (Eugenol) release via long-term EIS measurements was also carried out. For this reason, samples were soaked for 22 days in Hank’s solution.

(a) Determination of the optimal Eugenol concentration to be impregnated in TiO_2_.

**Figure 2:**
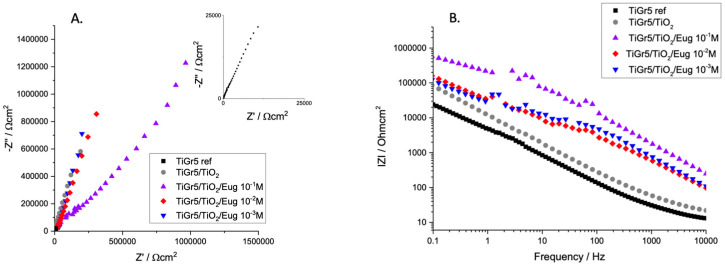
Nyquist (**A**) and Bode magnitude (**B**) EIS spectra of TiGr5 reference, TiGr5 coated with undoped TiO_2_, and with Eug impregnated coatings, TiO_2_/Eug 10^−1^ M, TiO_2_/Eug 10^−2^ M and TiO_2_/Eug 10^–3^ M.

Figure 2 shows the EIS spectra of bare TiGr5, and this material is coated with undoped TiO_2_ and Eugenol-impregnated layers (TiO_2_/Eug) in solutions of different Eug concentrations.

It is worth mentioning that titanium and its alloys do not typically present perfect capacitive loops (semi-circles) in their Nyquist EIS spectra. Previous studies attributed the phenomenon to surface roughness or multiple time constants on the same frequency range [42]. For this reason, these graphs are typically evaluated according to the steepness of the Nyquist spectra. It can be observed that an almost linear relation between the real and imaginary parts of impedance in Nyquist diagrams occurs in all cases; however, the plain TiO_2_ coating presents higher and steeper impedance spectra than that of the bare TiGr5 reference substrate. Eug-impregnated coatings show steep capacitive behavior. 

Bode diagrams usually present a more accurate and easy-to-interpret result. In the present case, the Bode diagram accurately depicts the conclusions drawn from the Nyquist diagram. The high values of |Z| at low frequencies are typical of good barrier materials. It indicates that the film formed on the samples is corrosion-resistant. Thus, judging by the values of the aforementioned coating impedance, TiO_2_/Eug 10^−1^ M was deemed an adequate concentration for improved anticorrosive behavior. Moreover, because this study aimed to produce effective antimicrobial coatings, 10^−1^ M was the chosen concentration to be introduced into the coating matrix in all further measurements to ensure the best results in both investigation areas.

(b) Long-term EIS measurements

**Figure 3:**
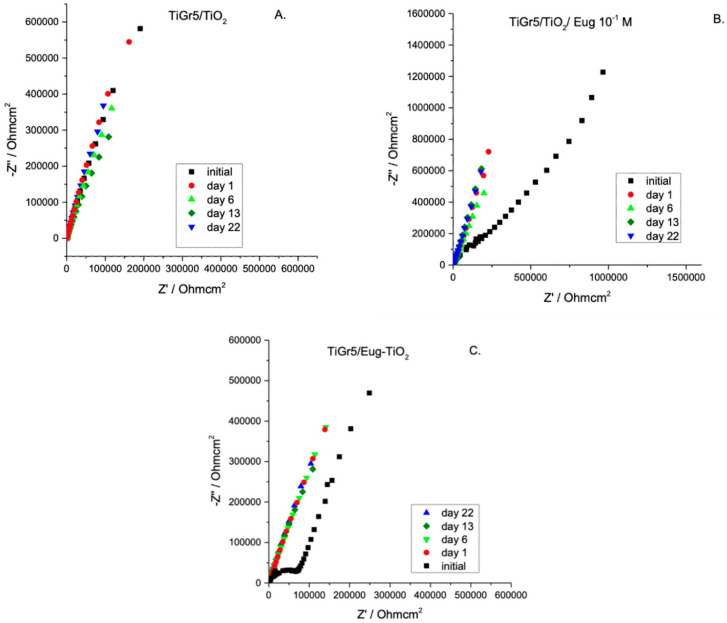
Nyquist EIS spectra of TiO_2_ (**A**), TiO_2_/Eug 10^−1^ M (**B**), and Eug–TiO_2_ (**C**) over a period of 22 days.

Undoped TiO_2_, TiO_2_/Eug 10^−1^ M, and Eug–TiO_2_ coatings on TiGr5 were measured in Hank’s simulated physiological solution over a period of 22 days to evaluate coating stability, resistance to corrosion, and additive release. Nyquist impedance spectra are presented in Figure 3.

**Figure 4:**
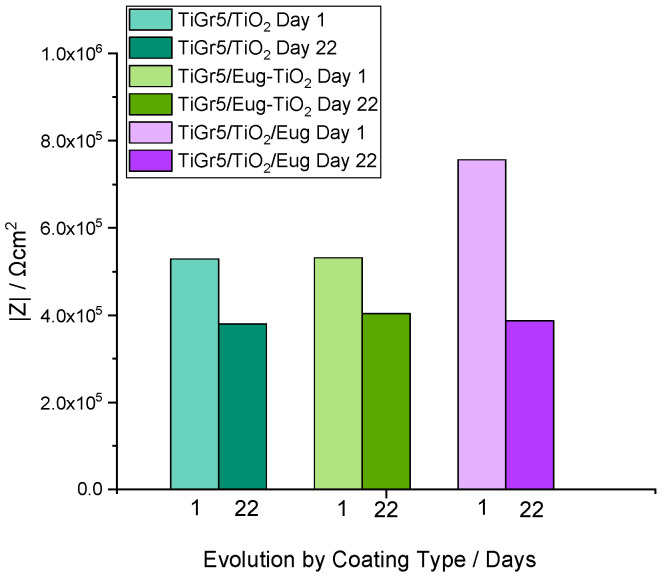
Histogram depicting the impedance modulus values of TiO_2_, Eug–TiO_2_, and TiO_2_/Eug on Day 1 and Day 22 of the long-term evaluation.

As expected, different stabilities of the investigated samples are noticeable. For all samples, the impedance modulus decreases over time. After 22 days of immersion, the curves for undoped TiO_2_ show a relatively small decrease indicating high stability (Figure 3A,B). Figure 4 compares the impedance modulus values of the first and last days for all coating types. The histogram clearly shows higher |Z|_0.01_ values of TiO_2_/Eug compared to the undoped TiO_2_ and the Eug–TiO_2_ coatings but show a steeper decline in resistance by the 22nd day. The undoped TiO_2_ and the Eug–TiO_2_ coatings show similar |Z|_0.01_ values, both lower than the impregnated coatings but with a slighter long-term decrease in value and resistance. The numerical representations of the decrease of |Z|_0.01_ values after 22 days were also determined and are as follows: ~28.1% in the case of TiO_2_, ~48.8% in the case of TiO_2_/Eug, and only ~22.5% for TiO_2_–Eug coatings. The steep concentration decline in the case of the impregnated TiO_2_/Eug coatings can be attributed to the mere adsorption-desorption phenomena, which does not occur in the case of the Eug–TiO_2_ system, and thus the coatings present higher resistance in long-term analysis. Even if the impedance modulus values of Eug–TiO_2_ are close to that of TiO_2_/Eug, the Eug–TiO_2_ coatings show an improved tendency on day 22, which suggests a better corrosion resistance. This leads to recommending the addition of Eug in the sol phase before dip-coating as a better practical solution than simple immersion in Eug solutions after dip-coating. 

**Figure 5:**
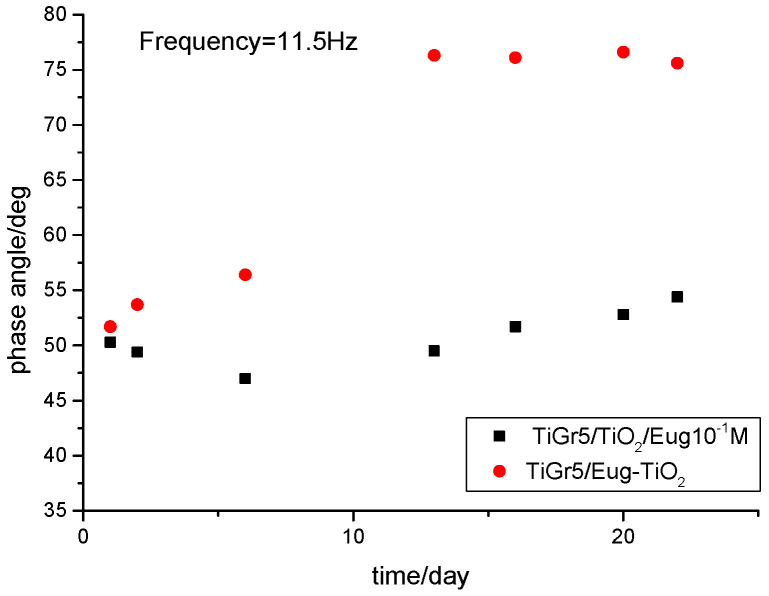
Comparison of phase angles at middle frequency domains forTiGr5/TiO_2_/Eug 10^−1^ M (◼) and TiGr5/Eug–TiO_2_ (●).

The electrochemical behavior of the different coating types was also investigated via the variations of phase angles in the middle-frequency domains of EIS. It was reported that in some cases, the variation of phase angles at 10 Hz with the immersion time was very close to the variation of coating resistance, hence may qualitatively reflect the coating performance [43]. As can be seen in Figure 5, a very small variation of phase angle is noticed in the 5–50 Hz frequency range during 22 days of immersion, pointing to good stability of the TiGr5/TiO_2_ 10^−1^ Eug samples. In the case of TiGr5/Eug–TiO_2_, in the early stage, a small variation of the phase angles is observed, but after 16 days, the values become very stable in the 5–50 Hz frequency range reaching higher values (around 75°) than in the case of TiGr5/TiO_2_ 10^−1^ Eug samples (around 55°).

Surprisingly, with the increase of immersion time, the phase angles for TiGr5/Eug–TiO_2_ measured at middle frequencies increase significantly, suggesting an increased resistive behavior of the coating.

**Figure 6:**
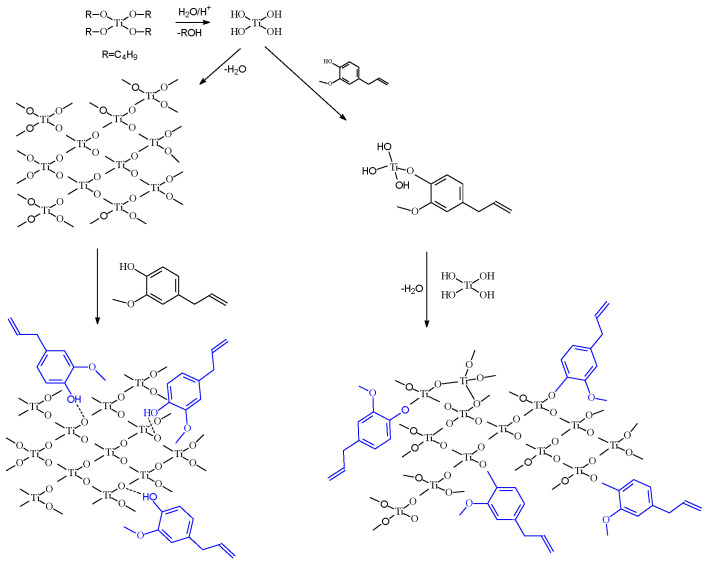
Scheme of the possible incorporation path of Eugenol into the TiO_2_ matrix.

A possible explanation for the better performance of the Eug–TiO_2_ coatings could be the incorporation of Eugenol in the TiO_2_ sol-gel layer by stronger interactions during condensation than in the case of simple immersion, where physical interactions are most plausible. The condensation reaction continues after the introduction of the samples in Hank solution, and the process leads to consolidated coatings after several days. In the presence of Eug, the precursor reactions in the sol-gel process are depicted in Figure 6.

TiO_2_ sol preparation is typically based on a hydrolysis-and-condensation reaction pair. In the presence of Eug in the precursor sol, it is presumed that the same reactions occur, with the inclusion of Eugenol by ether bonding.

Although the anticorrosive effects of Eugenol are not impressive, it was expected that this compound would have a beneficial contribution to the antibacterial properties of the coatings.

(c) Influence of the substrate

**Figure 7:**
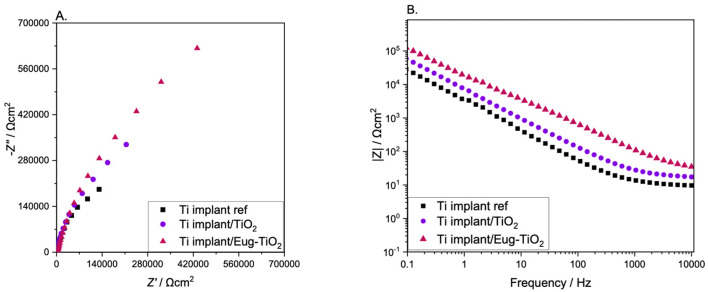
(**A**) Nyquist and (**B**) Bode EIS diagrams of Ti implant reference, Ti implant/TiO_2_, Ti implant/Eug–TiO_2_.

In order to see if the nature of the substrate influences the electrochemical behavior of the different coatings, in the next step, undoped TiO_2_, TiO_2_/Eug, and Eug–TiO_2_, in the previously analyzed concentration of 10^−1^ M, were used to coat an industrial piece cut from a Titanium implant (cp-Ti). The impedance measurement results are depicted in Figure 7.

It can be observed that the Ti implant used as a reference substrate presented the lowest impedance values, while the Ti implant/Eug–TiO_2_ showed the highest values, surpassing the Ti implant/TiO_2_. While the steepness has stayed almost the same in all three systems, the beginning of the semi-circle gained enough contour to lead to a conclusion based on the highest Z absolute values. In the present case, it can be concluded that Eugenol positively affected the anticorrosive behavior of the TiO_2_ coating on the Ti implant wafers, surpassing the layers formed on the TiGr5. This can be attributed to the different surface properties of the medically pre-treated Ti implant as opposed to the bare TiGr5, which had not undergone the series of pretreatments necessary for medical use, only for coating application.

#### 2.1.2. Potentiodynamic Polarization Curves

Potentiodynamic polarization curves were recorded to determine the layers’ corrosion rate and other specific kinetic and thermodynamic parameters. All types of produced coatings were subjected to polarization tests. 

Table 1 contains the kinetic parameters of measured polarization curves obtained by the Tafel interpretation method.

As expected, the results show that corrosion current densities on both TiGr5 and Ti implant substrates are lower when the metals are coated. On both substrates, Eug–TiO_2_ coatings have a protective role and present the lowest corrosion currents as compared to the bare reference metals, which means they exhibit the highest corrosion resistance. The effect is stronger when the substrate is the Ti implant, which may be due, as previously mentioned, to the different pretreatments applied at the production of implants, which could provide a better adherence of the coating, or simply better mechanical properties of the metal itself. In the case of both substrates, the introduction of Eugenol into the TiO_2_ matrix resulted in a more resistive coating than in the case of the undoped TiO_2_.

### 2.2. Fourier-Transform Infrared Spectroscopy

**Figure 8:**
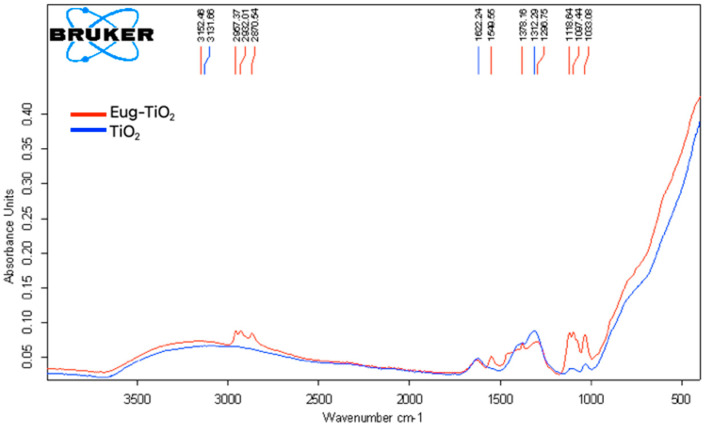
Overlay of FT–IR spectra of TiO_2_ (blue) and Eug–TiO_2_ (red) nanoparticles obtained from sol drying at 150 °C.

To explain the favorable behavior of Eug–TiO_2_ coatings and to put in evidence the possible interactions taking place between TiO_2_ and Eug, FT-IR spectra (Figure 8) of powders prepared from TiO_2_ and Eug–TiO_2_ sols were analyzed.

The FT–IR spectra of TiO_2_ (blue) and Eug–TiO_2_ (red) powders were recorded in the 400–4000 cm^−1^ range to determine whether specific bonds were formed between the additive introduced into TiO_2_. The particular bonds of TiO_2_ have been discussed in the literature, with stretching bonds at 690.52 cm^−1^ corresponding to Ti-O bonds [44]. In our case, for pure TiO_2_, a sharp, broad peak between the wavenumbers of 800 and 400 cm^−1^ can be identified, overlapping the peak mentioned above (see the blue curve). Another source in the literature attested that Ti-O-Ti bonds typically appear in the range of 820–763 cm^−1^ [45].

The FT–IR spectrum of Eug–TiO_2_ nanopowder was also recorded. According to a previous study [46], the specific values of Eugenol appear at about 2929 cm^−1^, corresponding to the stretching vibration -OH; 3000–2700 cm^−1^ wavenumber correspond to C-H stretching vibration; the peaks specific to the groups C=C can be observed between the values of 1550 and 1200 cm^−1^, with C-O connection, present between 1320 and 1030 cm^−1^.

In the case of Eug–TiO_2_ powder, specific peaks of both Eugenol and TiO_2_ have been identified. Additionally, Ti-O-C bonds [47], which confirmed the chemical bond between TiO_2_ and Eugenol, were identified as small peaks at values between 700 and 600 cm^−1^, almost shaded by the sharp, wide tip of Ti-O-Ti values which showed up in the interval of 450–820 cm^−1^. The existence of these interactions between Eugenol and TiO_2_ corroborates with the beneficial behavior of the Eug–TiO_2_ coating regarding corrosion resistance.

### 2.3. Raman Spectroscopy

**Figure 9:**
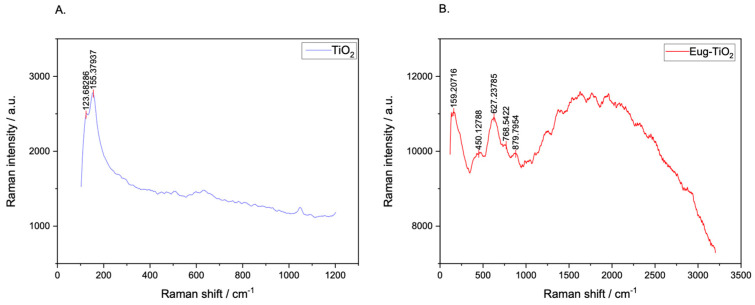
Raman spectra of TiO_2_ (**A**) and Eug–TiO_2_ (**B**) powders dried from corresponding sols.

The main scope of performing Raman spectroscopy measurements was to put in evidence specific Ti-O-C bonds, which, as mentioned before, show up on FT–IR as small peaks within the 700–600 [35] value range, being unfortunately overshadowed by the large, sharp peak of Ti-O-Ti. For this purpose, Raman measurements were effectuated to aid the investigation. Figure 9 depicts the recorded Raman spectra of TiO_2_ and Eug–TiO_2_. The presence of the aforementioned Ti-O-C bonds was confirmed by a small, slightly wide peak showing up at 627.23 cm^−1^, which cannot be seen on the undoped TiO_2_ powder. The results confirm that Eugenol does indeed incorporate in the TiO_2_ matrix via the presumed etheric bonding.

### 2.4. Antimicrobial Analysis 

An *E. coli* strain was used in the investigation to determine antibacterial properties. Its use is argued in various studies given its relative simplicity [48], as well as fast, high-density cultivation and thoroughly studied genetics [49], making it an appropriate bacteria to be used as an experimental model for all kinds of antibacterial studies.

The antibacterial efficacy of Eugenol doped TiO_2_ layers was tested to determine whether the studied coatings showed a propensity for medical use. The presence of compounds having antimicrobial properties on the surface of implants is vital to develop efficient methods for the prevention of bacterial infections and inflammations and even to relieve pain.

**Figure 10:**
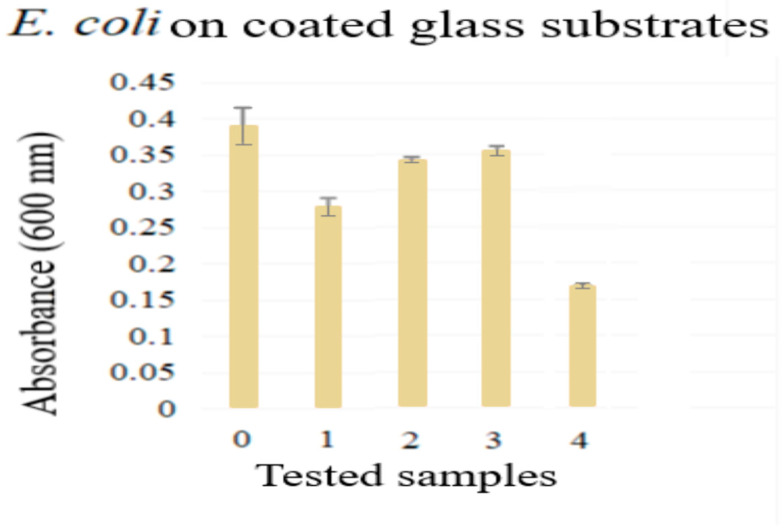
Antimicrobial analysis results for 0. blank sample, 1. TiO_2_, 2–3. TiO_2_/Eug in the following concentrations 10^−1^ M, 10^−2^ M, and 4. Eug-TiO_2_.

Microbiological test results are shown in Figure 10. All samples had different levels of inhibitory capacities against the *E. coli* strain used. The TiO_2_-coated glass sample had a mild inhibitory capacity as compared to the TiO_2_/Eug coated glass samples (2 and 3), while Eug–TiO_2_, the sample where Eugenol was directly incorporated into the TiO_2_ sol (4), was the most efficient against *E. coli*.

The antibacterial effects of Eugenol [50,51,52,53,54] and TiO_2_ [55,56,57] are well documented. The impregnated samples (2–3), as seen in Figure 10, showed a higher absorbance, meaning that there was a higher strain spread documented. This could be explained by the lack of chemical bonding between the two compounds (as impregnation leads to a physical bonding), which causes a weaker antibacterial effect due to the quick dissolution of Eugenol in the analysis medium. The samples of Eugenol incorporated into the TiO_2_ sol (which corresponds to the lowest absorbance) provided the best antimicrobial effect, since these samples had the lowest *E. coli* spread value compared to the other analyzed samples. This result might indicate a longer-lasting, synergistic effect, caused by the previously discussed bonding between Eugenol and TiO_2_.

### 2.5. Coating Adhesion Tests

Adhesion presents an essential parameter for coating performance. The better the coating adhesion, the more unlikely it is that electrolyte or other corrosive substances make their way underneath the layer and onto the metal surface to start an even more aggressive, localized corrosion process. Adhesion can typically be measured by international standards [58], or percentage-wise with the aid of the Lattice–Notch formula. The Lattice–Notch method is based on the determination of the coating adhesion by the determination of the ratio between the total amount versus the ripped-off number of squares in the square mesh. Equation (1) describes the mentioned correlation.

(1) $$Adhesion=\frac{a-b}{a}\times 100$$

where a denotes the total amount and b stands for the ripped-off number of squares from the engraved square mesh on the surface of the coated metal substrate.

It was concluded that all the coating types analyzed presented a spectacular 99.9% adhesion.

### 2.6. Coating Thickness Evaluation

Corrosion resistance is greatly influenced by another physical parameter: the coating thickness. Its awareness can contribute to a smoother understanding of the occurring electrochemical phenomena. Coating thickness performance, whether high or low, greatly depends on the coating materials used. While some coatings produce better results with a high coating thickness, others may prove to be more performant with nano-scale coatings.

In the present study, the aim had been to produce layers adequate for medical use. Table 2 presents the average coating thickness values of all measured layers. It can be seen in Table 2 that impregnated coatings TiO_2_/Eug presented the highest thickness value, most probably due to the formation of a loose layer of Eug physically adsorbed on the TiO_2_ surface. On the other hand, Eug–TiO_2_ coating prepared by introducing Eugenol in the precursor sol showed a slight decrease in coating thickness, as compared with undoped TiO_2_, most probably due to a slight contraction of the coating as a result of the chemical bonds formed between Eug and TiO_2_. All coatings proved to be micrometer thin, and the thicknesses were the same on both TiGr5 and Ti implant substrates.

### 2.7. SEM Measurements

**Figure 11:**
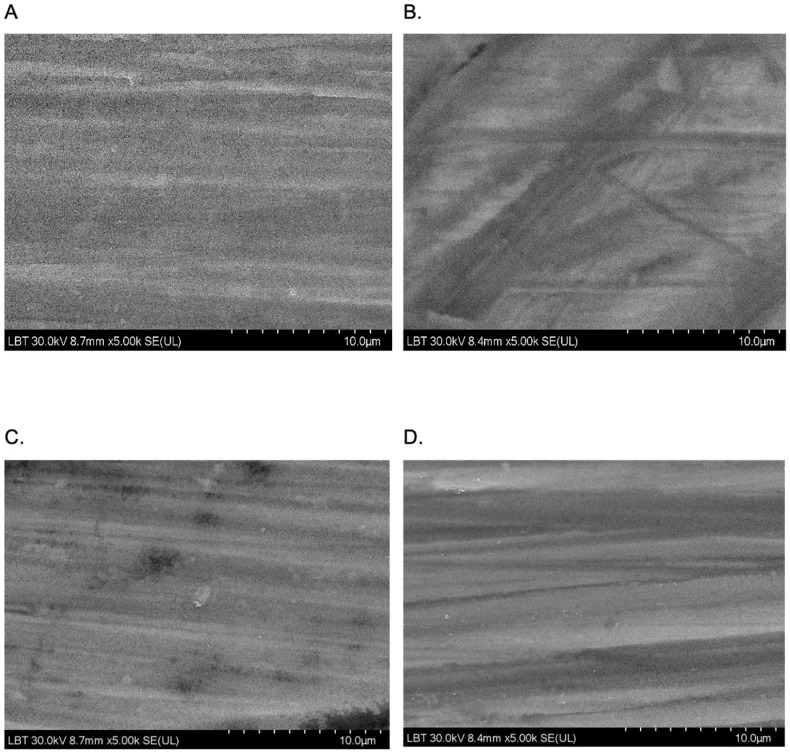
SEM images were recorded at a magnitude of 10 µm on TiGr5/TiO_2_ (**A**), TiGr5/Eug–TiO_2_ (**B**), Ti implant/TiO_2_ (**C**), and Ti implant/Eug–TiO_2_ (**D**).

SEM analysis was performed in order to obtain an insight into the surface morphology of the prepared coatings, namely TiO_2_ and Eug–TiO_2_, on both TiGr5 and Ti implant substrates. The micrographs are presented in Figure 11. It can be observed on all recorded images that there neither in the case of the Eug–TiO_2_ and TiO_2_ coatings nor in the case of the two bare substrate types (TiGr5 and Ti implant), there is no perfect covering of the surface, so in both cases, the substrate roughness remains visible. As the coating thicknesses are very close, it can be concluded that the intrinsic characteristics of the coatings are responsible for the different corrosion behavior of the samples.

**Figure 12:**
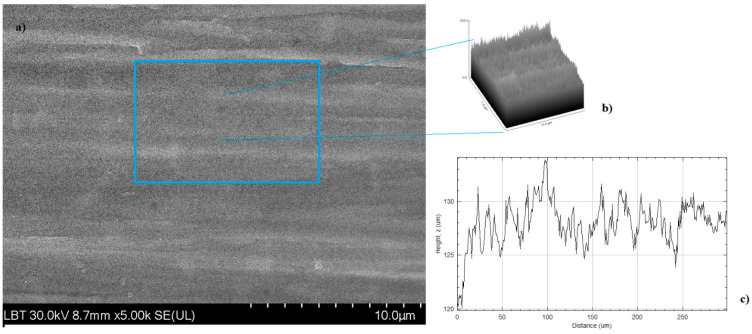
2D SEM image recorded at a magnitude of 10 µm on TiGr5/TiO2 (**a**) 3D model (mesh and texture of intensities) of sample TiGr5/TiO2 (**b**) R-profile of the surface (**c**).

The roughness of the surface was calculated using ImageJ software (version 154f) for image processing and analysis of the surface (Figure 12). 

By performing these image analyses, quadratic mean roughness Rq and individual variances of the peaks and valleys average (Ra) parameters were determined. The results are presented in Table 3.

From the above-mentioned parameter values in Table 3, it can be seen that, in the case of both substrates, with the incorporation of Eugenol in the TiO_2_ coating, its unevenness decreases, and the surface becomes smoother.

## 3. Conclusions

TiGr5 and Ti implants were used as metal prototypes for the application of TiO_2_ sol-gel coatings doped with Eugenol by two methods, namely by impregnation after deposition of TiO_2_ and by direct mixing into the TiO_2_ precursor sol. 

Electrochemical evaluation of prepared coatings led to the conclusion that Eugenol does not improve the anticorrosion behavior of TiO_2_ coatings, when introduced by impregnation. On the contrary, layers produced by direct introduction into the precursor sol during TiO_2_ coating preparation generated promising impedance values, well above those of the impregnated coatings, on both metal substrates. Ti implant substrate proved to be more adequate, presumably because of the different industrial production methods and pretreatments applied on medical ware, compared to industrial provenance grade V Titanium (TiGr5).

The Fourier-transform infrared spectroscopy (FT–IR) analysis showed evidence of bonding between Eugenol and TiO_2_ by the appearance of specific Ti-O-C bonds on the recorded spectra. 

Raman spectroscopy analysis was performed to confirm Ti-O-C, which was barely seen on FT-IR images. Specific Ti-O-C bond peaked at 627 cm^−1^, confirming the chemical bonding between Eugenol and TiO_2_ when directly introduced into the precursor sol.

Antimicrobial analysis performed by the cultivation of *E. coli* on all types of investigated Eugenol-doped coatings showed significant antimicrobial activity added to Eugenol’s known analgesic and anti-inflammatory effects.

Adhesion and coating thickness evaluations were also performed. Adhesion of all types of applied coatings independently resulted in a 99.9% adhesion which is favorable for medical wear. Also, coating thickness tests proved that all coating types were of micrometer thickness.

SEM analysis was performed on TiO_2_ and Eug–TiO_2_ coatings on both TiGr5 and Ti implant substrates. The recorded SEM images showed smooth and clear coating surface morphology in each studied case.

Given the results, it can be concluded that TiO_2_ coatings improved with Eugenol by mixing it into the precursor sol are promising coating prototypes on both TiGr5 and Ti implants, with antimicrobial, anti-inflammatory, and analgesic properties, as well as unshaken anticorrosive effects. 

## 4. Materials and Methods

### 4.1. Materials

Two Ti-based metallic substrates were used: Ti6Al4V (TiGr5) and one specific cp-Ti implant substrate (Sanatmetal RO SRL), which is a 99.8% Ti alloy also containing iron (Fe-0.060), oxygen (O-0.140), nitrogen (N-0.004), hydrogen (H-0.003), and carbon (C-0.016).

Sulfuric Acid (H_2_SO_4_, 96%, Carlo Erba, Milan, Italy), Isopropyl Alcohol (99.7%, Chemical Company), Acetone (Reagent for Analysis, Chemical Company, Iasi, Romania), Ethyl Alcohol (EtOH, Absolute, Denatured, Reagent for Analysis, Chemical Company) and distilled water were used for the pretreatment of TiGr5, cp-Ti implant, and glass substrates.

Sodium chloride (NaCl, 99.5%, Chemical Company), Calcium chloride (CaCl_2_, 97.5%, Sc. Nordic Invest SRL), Potassium chloride (KCl, reagent for analysis, 99.5%, Merck), Sodium bicarbonate (NaHCO_3_, reagent for analysis, Merck KGaA, Darmstadt, Germany), Sodium phosphate monobasic monohydrate (NaH_2_PO_4_·H_2_O, >99.0%, Sigma–Aldrich, Darmstadt, Germany), Sodium phosphate dibasic dihydrate (Na_2_HPO_4_·2H_2_O, >98.0%, Sigma-Aldrich), Magnesium chloride hexahydrate (MgCl_2_·6H_2_O, 99.0%, Chemical Company), Magnesium sulfate heptahydrate (MgSO_4_·7H_2_O, reagent for analysis, 99%, Chemical Company), D (+)-glucose monohydrate (C_6_H_12_O_6_·H_2_O, >99.0%, Merck) and distilled water were used to prepare Hank’s simulated physiological solution as per the literature [29].

Titanium (IV), N-butoxide (TNB, 99 +%, Alfa Aesar, Tewksbury, MA, USA), Ethyl Alcohol (EtOH, absolute, denatured, reagent for analysis, Chemical Company), and Nitric Acid (HNO_3_, 65%, Merck) were used as raw materials for the synthesis of TiO_2_ precursor soil.

Eugenol (100% eugenol oil, CERKAMED Medical Company, Stalowa Wola, Poland ) was introduced into the TiO_2_ layers. 

Distilled water was used to prepare each solution. All chemicals were of analytical grade and used without further purification.

### 4.2. Preparation of the Precursor Sols

**Figure 13:**
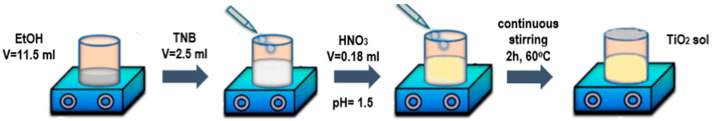
Scheme of the preparation of the pure TiO_2_ sol step-by-step.

- Titanium (IV) N-butoxide (TNB) was used as a precursor for the pure TiO_2_. A total of 2.5 mL of TNB was dissolved in 11.5 mL ethanol (EtOH) at room temperature with continuous stirring. The pH of the precursor sol was adjusted to 1.5 by adding 0.18 mL of nitric acid (65% HNO_3_). The solution was stirred for 2 h at 60 °C [42]. A basic step-by-step scheme of the TiO_2_ sol preparation can be seen in Figure 13;
- Coating impregnation happened by immersing the TiO_2_-covered TiGr5 plate into an alcohol-based, Eugenol-containing solution of different concentrations (10^−1^ M, 10^−2^ M) for 30 min each;
- For the Eugenol-containing sol, Eugenol was added directly into the solvent (in the pre-determined optimal concentration, following coating impregnations), after which the sol was prepared the same as mentioned in part A.

### 4.3. Coating of the Metal Substrates and Glass Plates by Dip-Coating Method

Both metal (TiGr5 and cp-Ti implant) and glass substrates were subjected to a thorough pretreatment process prior to the application of TiO_2_ sols. Metal substrates of 1 mm × 15 mm × 20 mm were polished with emery paper (P280, P400, P600, P800, P1000, P2000, P5000) and then rinsed with distilled water. Lastly, the metal substrates were sonicated in an ultrasonic bath, and immersed in acetone and ethanol for 10 min each. 

The microbiological analysis was performed on glass plates. The glass plates were first rinsed with distilled water, then washed with a perfumeless detergent, sulphuric acid aqueous solution (10 wt% H_2_SO_4_), and isopropyl alcohol.

Metal and glass substrates alike were coated using the dip-coating method at 12 cm/min withdrawal speed. Four types of coatings were applied to metal surfaces: pure TiO_2_, Eugenol-impregnated TiO_2_ (10^−1^ M, 10^−2^ M), labeled TiO_2_/Eug, and Eugenol containing TiO_2_ coatings, prepared by direct adding Eug to the precursor sol, labeled Eug–TiO_2_. The bare TiO_2_ and Eug–TiO_2_ were also used to coat a Ti implant substrate. The layers underwent a thermal treatment of 150 °C for one hour after dip-coating in an oven. 

### 4.4. Electrochemical Characterization of TiO_2_ Coatings

Electrochemical measurements were performed on an AUTOLAB PGSTAT302N (Metrohm) potentiostat in a three-electrode cell containing a working electrode (TiGr5 plate and Ti implant wafers), a counter electrode (Pt plate), and a reference electrode (Ag/AgCl/KCl_satd_). Hank’s simulated physiological solution was used as an electrolyte [59]. 

Open circuit potential measurements were performed in each case for 1 h. Electrochemical impedance measurements (EIS) were performed in the frequency range 10 mHz–10 kHz, with a sinusoidal current of 10 mV amplitude, at the OCP value. At last, the potentiodynamic polarization curves (PDP) were recorded between OCP ± 20 mV and OCP ± 200 mV, the latter being intentionally left as the last operation, as great overvoltage damages thin films.

### 4.5. Microbiological Evaluation of Coated Glass Substrates

Antibacterial activity was tested against Gram-negative bacteria *Escherichia coli* (*E.coli*) (ATCC 25922). For these tests, glass slides were coated as mentioned in Section 2.4, after which the samples were prepared according to EUCAST protocols [60]. Briefly, the covered glass slides with the applied treatments were left under UV light for 10 min for decontamination and then placed in 12-well plates in Nutrient broth media (VWR Chemicals, VWR International GmbH, Wien, Austria). At a confluence of 0.5 McFarland turbidity, the bacterial strain was added to the wells (10 µL at 1 mL media) and left for 24 h at 35 °C to develop. After this interval, the liquid was placed in a 96-well plate, and the optical density was read at 600 nm using an EPOC BioTek spectrophotometer (BioTek Instruments, Winooski, VT, USA). The results were compared to the untreated control.

### 4.6. Adhesion Tests

Adhesion tests of applied coatings were performed using a TQC Adhesion Test KIT. The tests were performed by first cutting a 7 × 7 mm square mesh into the surface of the layer. Special duct tape was then placed on top of the cut square mesh, then ripped off with one swift, decisive move. The adhesion was then determined by the Lattice–Notch method, based on the quotient of squares left on the surface subtracted from the total number of squares and then divided by the total number of squares and multiplied by 100. The result is a percentile characteristic of the measure of coating adhesion. The test was effectuated on all produced coating types. Adhesion classification was also made based on international standards.

### 4.7. Coating Thickness Evaluation

Coating thickness measurements were performed on all types of coatings produced. The measurements were effectuated with a TROTEC BB25 instrument by placing the instrument perpendicularly on the surface of the layer and gently pushing it down until the apparatus displayed the thickness value. Measurements were based on magnetic induction and turbulent flow law and were repeated several times on the same sample. The mean value of repeated measurements was considered. The device offered 0.1% precision. 

### 4.8. Fourier-Transform Infrared Spectroscopy

Fourier-transform infrared spectroscopy (FT–IR) measurements were performed on a BRUCKER ALPHA II PLATINUM- ATR on a 400–4000 cm^−1^ wavelength scale. TiO_2_ and Eug–TiO_2_ sols were placed in a drying oven in a beaker at 150 °C for curing until the leftover solvent (EtOH) evaporated, then powdered and measured in the solid phase.

### 4.9. Raman Spectroscopy

Raman spectroscopy analysis was performed on a Renishaw inVia Raman spectrometer and Leica microscope—the analysis aimed to indicate specific Ti-O-C bonds which Ti-O-Ti possibly overshadowed in FT-IR spectra. Measurements were carried out on TiO_2_ and Eug–TiO_2_ powders previously dried from the above-mentioned sols.

### 4.10. Scanning Electron Microscopy Analysis

Scanning electron microscopy (SEM) analysis was performed in order to investigate the coating surface morphology of the TiO_2_ and Eug–TiO_2_ coatings on both TiGr5 and Ti implant substrates. SEM measurements were made using a Hitachi SU8230 ultra-high resolution scanning electron microscope.

## Figures and Tables

**Table 1:** The kinetic parameters of the corrosion process in the case of TiGr5, TiO_2_, Eug–TiO_2_, and Ti implant ref, Ti implant/TiO_2_, Ti implant/Eug–TiO_2_.

Sample	i_corr_ [A/cm^2^]	b_c_ [V/dec]	b_a_ [V/dec]	E_corr_ [V]	v_corr_ [mm/Year]
TiGr5 ref	1.19 × 10^–8^	0.052	0.084	–0.379	1.775 × 10^–4^
TiGr5/TiO_2_	1.49 × 10^–8^	0.038	0.146	0.019	1.728 × 10^–4^
TiGr5/Eug–TiO_2_	4.30 × 10^–9^	0.087	0.023	–0.055	4.991 × 10^–5^
Ti implant ref	6.98 × 10^–8^	0.116	0.110	–0.306	1.041 × 10^–3^
Ti implant/TiO_2_	6.81 × 10^–9^	0.119	0.097	–0.255	1.016 × 10^–4^
Ti implant/Eug–TiO_2_	5.37 × 0^–10^	0.061	0.068	–0.054	8.012 × 10^–6^

**Table 2:** Coating thickness values of all produced layer types.

Sample	Thickness [µm]
TiO_2_	96 ± 0.1
TiO_2_/Eug	127 ± 0.1
Eug–TiO_2_	91.5 ± 0.1

**Table 3:** Rq and Ra parameters for the samples.

No.	Name	Rq	Ra
1	TiGr5/TiO_2_	42.3781	17.4778
2	TiGr5/Eug–TiO_2_	30.7384	10.9787
3	Ti implant/TiO_2_	39.8148	15.5916
4	Ti implant/Eug–TiO_2_	35.5872	13.2787

## Data Availability

Not applicable.
